# Historical perspective fifty years of particles: a personal retrospect

**DOI:** 10.1186/1743-8977-8-35

**Published:** 2011-12-29

**Authors:** Anthony Seaton

**Affiliations:** 1 Emeritus Professor of Environmental and Occupational Medicine, The University of Aberdeen, King's College, Aberdeen, AB24 3FX; 2 8 Avon Grove, Edinburgh EH4 6RF, Scotland, UK

## 

The year 2010 was the 350^th ^anniversary of the foundation of the Royal Society of London, the world's oldest scientific academy. Among its earliest correspondents was the Dutch instrument maker, Anton van Leuwenhoek, whose single lens microscopes opened the eyes of the world to what had previously been invisible. In that same era of scientific and philosophical advance, the Enlightenment, attention was first drawn to the problem of industrial pollution by John Evelyn's petition, *Fumifugium*, to King Charles II in 1661. However, it was not until late in the Industrial Revolution, and the rise in use of coal as a source of power in factories and heating in homes, that the full impact of particulate pollution began to be recognised. Pollution episodes in Donora, Pennsylvania and the Meuse valley in Belgium in the 1940s, and the great London smog of 1952, were associated with dramatic increases in deaths in the local populations and led eventually to Western Governments taking action to curb emissions. Nevertheless, coal remained the primary cause of urban pollution into the 1970s, when oil began to take over.

## Early days

My first conscious meeting with particles was in the 1940s as a child in Liverpool and Leeds, two heavily polluted UK cities where cold, still winter days were characterised by dense smogs that prevented one seeing across the street. In the former city during the early part of the 2^nd ^World War, matters were temporarily worsened by the generation of smoke screens across the city to obscure it from the bombers of the Luftwaffe, the coal ovens being sited across the street from my parents' apartment. It was an ineffective measure and the bombs continued to fall! As a medical student in the 1950s I became aware of the fact that these smogs were associated with large numbers of excess deaths, and every winter the hospital wards in that era filled with patients in respiratory failure; smoking was almost universal in adults and chronic bronchitis was known then as the English Disease, fully justifying King James VI of Scotland's famous Counterblast to Tobacco of 1604; "*A custom loathsome to the eye, hateful to the nose, dangerous to the lungs..*." But so often a pollution episode provided the *coup de grâce*. Public health measures, in the UK the Clean Air Acts, gradually reduced this black smoke pollution and in Western Europe the effects became much less obvious - we began to believe that urban air pollution was no longer a problem, especially as it was much less easy to see, and the haziness of the air in large cities began to be accepted as normal. The pollution not only had been reduced but also, subtly, it had started to change colour. What had been measured as British Black Smoke, a relatively crude but useful colorimetric method, was now measured as PM_10 _or PM_2.5_, particulate matter less than 10 or 2.5 micrometers in aerodynamic diameter. By one of those ironies that are so familiar in academic life, the pioneering air pollution research unit at St Bartholomew's Hospital Medical School in London, which had made huge inroads into understanding and quantifying pollution under Prof Patrick Lawther, closed just as the new evidence from major US epidemiological studies of pollution began to be published.

My next meeting with particles was as a young doctor. In 1964 I spent a year working in Stoke on Trent, site of the famous Wedgewood pottery industry, and here I saw both men and women suffering from silicosis from exposure to quartz used in glazes and for bedding porcelain in the kilns. I learned of the efforts made to protect these workers and the studies that had shown the highest exposures often to have come from changing their clothes at the end of a shift. I learned that the disease had been known and understood pathologically since the late 18^th ^century, yet was still occurring and was untreatable - unsuccessful attempts had been made to ameliorate its effects by inhaling aluminium oxide powder, on the theoretical understanding that the surface of the quartz crystal became less toxic if it were coated with other minerals. I was interested to find that the pottery industry was sited in Stoke because of the local availability of coal, the china clay (kaolin) being brought there from Cornwall in the south west of England, initially by canal then by rail, and I also saw my first patients with coal worker's pneumoconiosis in that year. An interest in industrial medicine and in the prevention of industrial disease was born and, after some further forays into cardiology and neurology, I found myself researching pneumoconioses as a chest physician in West Virginia, USA in 1969.

## West Virginia, Cardiff, coal and Aspergillus

In the early 20^th ^century West Virginia had been the scene of coal mine wars, when owners had fought the unions, quite literally, bringing non-unionised labour mostly from Eastern Europe, in trains equipped with armour plating and machine guns, to work in the mountain mines of the State. An uneasy truce existed when I was there, but still there were tensions, notably with respect to compensation for pneumoconiosis. A broadly sympathetic Federal Government had enacted a compensation law, the Black Lung Act, and doctors were cast in the role of deciding who should and should not get the money. Often, scientific evidence took second place to political judgement in making these decisions and I worked in a unit that was charged with accruing the evidence. The main issue was this; smoking causes chronic obstructive pulmonary disease (COPD), most miners smoked and many get COPD, coal causes pneumoconiosis but pneumoconiosis is not clearly associated with COPD. In many miners the two diseases coexist, but does coal dust *per se *cause COPD? Some of our preliminary studies suggested that it did [1,2], but there was a lot of opposition to the idea, based on misleading epidemiology. No-one had at that time published data containing estimates of exposure to coal dust, but this was very shortly to change. In 1970 the Edinburgh Institute of Occupational Medicine (IOM) published a landmark paper in Nature on the association between coal dust exposure and risk of pneumoconiosis, based on careful measurements and estimates of dust exposure on some 30,000 miners [3].

At that time I was contemplating returning to the UK to work in the NHS as a full-time chest physician, and within a year I had obtained a post in Cardiff, South Wales, close to Britain's biggest coal mining area. The MRC pneumoconiosis unit there was then mainly interested in asbestos, Chris Wagner having arrived a year or two before from South Africa where he had described the association of mesothelioma with crocidolite exposure [4], but the unit's reputation was founded on the work it had done under John Gilson on standardisation of radiology and lung function for epidemiology in coal mining. It had never, however, investigated exposure assessment and had thus missed the opportunity which had been taken by the UK National Coal Board to investigate exposure-response relationships. My contact with the unit was limited, my main role at this stage being in the clinical management of asthma, though my interest in occupational disease led to me co-authoring a book on the subject with my US colleague, Keith Morgan [5]. For a few years my research interest was captured by another sort of particle altogether, *Aspergillus fumigatus *[6,7]. This common fungus lives in the soil on dead organic matter and its tiny spores, c3 μm in diameter, are responsible for an impressive number of human and animal respiratory diseases, unlike any other airborne fungus. What was it about this organism that conferred such pathogenicity? A series of studies later showed it had the power to resist phagocytosis by macrophages and indeed was able to turn the tables on phagocytes by immobilising them and using them as a food source [8]. This was clearly an advantage to survival from phagocytosis in the soil by competing organisms, and its pathogenicity was irrelevant to its life history, as it had never relied on colonisation of lungs to reproduce - a chance consequence of its need to survive in the soil.

## Edinburgh and the Institute of Occupational Medicine

This interlude of study of microbial particles proved inconclusive as we were never able to identify the chemical released from the spore surface that had this remarkable paralytic effect on motile cells, and to my knowledge this remains unsolved to this day. The studies overlapped my move in 1977 from Wales to Scotland and the Institute of Occupational Medicine; a return to mineral particles, since IOM had been founded in 1969 as a charitable research institute by the National Coal Board to research mining diseases.

In 1977, when I arrived, the work of the Institute was centred on coal and asbestos diseases. The coal research led by Michael Jacobsen was based on an epidemiological programme known as the Pneumoconiosis Field Research (PFR). Having first described the association between pneumoconiosis and respirable dust exposure, its emphasis was on refining these complex relations (how much and what kinds of dust cause pneumoconiosis?) and on the question raised above, does coal dust cause chronic obstructive lung disease? The key to the success of this research was the multi-disciplinary nature of the scientific effort - epidemiologists worked closely with physicians, physiologists, occupational hygienists, physicists, biologists and pathologists. The essential contribution had come from the physicists who asked the question: how can we measure the fraction of particles that reaches that part of the lung, the acinus, where pneumoconiosis develops? The answer was the invention by two physicists, Henry Walton and Bill Hamilton, of the MRE 114A respirable dust sampler, which was used in all the research as well as generally in coal mines to monitor dust levels [9]. This allowed quantification of the exposures of the miners to particles less than c7 μm aerodynamic diameter and estimation of the relationships between these and their risks of radiological change, functional impairment, pathological changes in the lung and mortality. It also allowed estimation of the effects of quartz in contributing to their health risks. Central to the work on lung pathology were John Davis and Anne Ruckley, who were finally able to show a clear relationship between coal dust exposure and pathological emphysema [10], a finding crucial to obtaining recognition that this disease (and the consequent COPD) was in fact a risk not only of smoking but also of coal dust exposure. This finding coincided with work led by Bill Marine which demonstrated the relationship of coal dust exposure with decrements in lung function consistent with obstructive lung disease [11].

This productive period from 1977 to 1990 also saw the IOM study a number of other particle exposures in a similar manner, all leading to the promulgation of UK and, in one case USA, Dust Standards. These were the wool industry, polyvinyl chloride production and the, by then defunct in UK, oil shale industry [12-14]. In all three cases relationships between exposure to the relevant dust and lung disease were demonstrated for the first time, something that makes me wonder how many other dusty industries are associated with damage to the lungs that has not yet been demonstrated. An old colleague used to say "the normal human is the one who has not yet been fully investigated!" There is some truth in this with respect to dusty trades also. The shale studies were of particular relevance to the possible increase in the use of shale deposits as a future source of oil, in view of the political instability of the major sources of this commodity.

As a chest physician I had witnessed the rise in lung cancer associated with smoking (the association was first described by Richard Doll when I was a student), and in my later years it was very satisfying to see a decline in this disease in men, though unfortunately it has now become the most common cancer in women as our relative smoking habits have changed. I saw my first patient with mesothelioma, an incurable malignant disease of the pleura, in 1971 at which time it was a very uncommon disease. By the time I retired in 2003 it was the cause of more than 2000 deaths annually in UK and I saw a patient with it almost every week. The cause was of course the increased exposure of workers to asbestos, especially amphiboles, from the 1930s onwards until about 1980 [15]. Sadly, this epidemic will continue for at least another decade. During my time in Cardiff I got to know Chris Wagner well and when I arrived in Edinburgh I found John Davis to be pursuing the same line of research. Although the cause was known, it was essential to prevention, as long as asbestos was used, to understand the mechanisms, and this acquainted me with particle toxicology. Again the IOM had the great advantage of having skilled physicists under Jim Vincent who could generate and measure dust clouds, toxicologists led by Ken Donaldson who could measure effects *in vivo *and *in vitro*, and epidemiologists including Brian Miller and Fintan Hurley who in helping to plan and analyse studies could make sure that all their conclusions were valid. It might be thought that the asbestos issue could have been solved simply by banning the material, but this does not take account of the exposures likely to occur when asbestos already in place is removed or disturbed. Nor does it take account of the need of industry for durable fibres which might have the potential to cause similar effects on the health of workers. This programme of work led to the validation of the important paradigm of fibre toxicity, showing much of the toxicity of fibres to lie in their length, diameter and durability in tissue [16]. This research also contributed to understanding that the durability of the fibres was critical to the causation of mesothelioma, explaining why the least durable, chrysotile, was a less potent cause than the amphibole types. Alongside this work, the IOM physicists designed an eyepiece microscope graticule incorporated in fibre counting rules for a UK Asbestos standard [17], and set up national and international quality control schemes for fibre counting.

By the late 1980s the nationalised UK coal industry was in decline and British Coal attempted to close the IOM, which by then had established a strong international reputation. After a considerable struggle, it proved possible to set it up in 1990 as an independent self-funding charity. It is particularly pleasing to see that it continues as a strong research institute to this day. The original key to the IOM's survival was persuading British Coal to make a one-off grant and by obtaining generous short-term research funding from the man-made fibre industry and the Colt Foundation. These grants, together with the retiral from IOM of the most senior staff provided the launch pad for its subsequent success under Colin Soutar's and now Phil Woodhead's leadership.

## Aberdeen, asthma and air pollution

I had the good fortune to obtain a part-time post in Aberdeen University in 1988 and this gave me the opportunity to move there when the IOM closed in 1990. I was also asked to chair the UK Government's Expert Panel on Air Quality Standards (EPAQS), in spite of having next to no expertise in the subject. But a move of job in one's 50s is stimulating and gives rise to new ideas. At Aberdeen I was freed from the burden of running a large research institute but faced with the different burden of starting my own research from scratch. I reverted to my clinical persona, and asked why asthma had increased so much during my professional lifetime. This was clearly an environmental effect and the smart money was on pollution or biological particles, even though neither had increased. A rather fanciful theory based on illogical grounds held that it was something to do with lack of infection or over-cleanliness. I felt that none of these was plausible and in any case held little prospect of leading to preventive action, so my colleagues and I proposed that although attacks of asthma may be provoked by inhaled biological or air pollution particles, the cause of the rise was likely to be a consequence of increased population susceptibility, perhaps from the rather dramatic changes in diet I had noticed in my lifetime [18]. I'm afraid that this idea was received with a deafening silence and it was obviously difficult to obtain research funding. However, we persevered and a programme of research into the effect of vitamin intake by the mother during pregnancy has now produced sufficient evidence of effects of vitamins D and E on early airway development for it to continue under my successor, Graham Devereux, some 20 years later [19,20]. Happily, our epidemiological results have now received support from similar studies in USA [21,22]. A broad lesson from this is that changes in the prevalence of disease must be environmental but susceptibility may change as well as the environment; susceptibility was always thought to be largely genetic but this is not so. Not only may genes be turned on or off by environmental factors such as infections or nutrition, but also the target tissue may be made more or less susceptible in the same way. Thinking laterally is very desirable in research. Equally important is finding the right colleagues and this work relied very much on the expertise of Graham Devereux, Geraldine McNeill, and a number of excellent PhD students. I hope that it will soon be possible to give pregnant women science-based advice on the best diet to reduce the risk of asthma in their offspring.

The work of EPAQS was another example of multidisciplinary collaboration, as the Panel included epidemiologists, clinicians, meteorologists and experts in environmental measurement. In the 10 years I chaired it we recommended science-based standards for nine pollutants, including particles. Among the early members was Robert Waller who had been one of the original members of the St Bartholomew's unit. He had made measurements of pollution in the old days of smog and was healthily sceptical of the findings of the more recent epidemiology. The concentrations of particles were so low compared to those in the 1950s that it seemed implausible that they could cause effects such as death from heart attack. He and others thought that there was a hidden confounder, probably related to inability to account adequately for temperature in the statistical models. The argument was that it was very unlikely that the inhalation of around a milligram of carbon over 24 hours could kill people, and I have to say that I agreed. Yet the epidemiology did indeed suggest strongly and consistently that this was the case [23,24]. Air pollution suddenly seemed interesting! A plausible explanation was necessary, one that could be tested experimentally, and it came to me by serendipity.

Following one such discussion of plausibility I went home and picked up the latest Lancet, which contained a paper on seasonal changes in fibrinogen levels in people's blood [25]. No explanation was offered other than possible infections, which were known to cause rises in fibrinogen. Fibrinogen is involved in the cascade of chemical changes that lead to blood clotting and blood clotting is known to be a factor in heart attacks (once called coronary thrombosis). Eureka! Maybe inflammation in the lungs caused by particles could induce rises in fibrinogen sufficient to increase the risk of a heart attack in susceptible people? But still there was the problem of the tiny dose of particles associated with this effect. I recalled the pioneering studies of Gunther Oberdörster and colleagues in which they had shown the different effects of ultrafine particles of titanium dioxide compared to larger particles of the same material [26] and I was aware of work dating back to the 1960s in London which had shown the huge numbers of such particles in air pollution [27]. Could it be that, not the mass, but the number of particles, mostly very small and capable of penetrating the respiratory epithelium, triggered interstitial inflammation and subsequent changes in blood clotting? My colleagues Ken Donaldson, Bill MacNee and David Godden and I wrote our hypothesis paper and it was published in the Lancet later that year, 1995 [28]. I think it is fair to say it changed the way scientists thought about and investigated air pollution and the effects of particles.

## Nanoparticles

Fundamental to the Lancet paper was the understanding from Oberdörster's group that what we then called ultrafine particles behaved differently in the lung to larger particles of the same material, having the ability to cross cell and tissue barriers and to cause greater inflammatory responses. Air pollution epidemiological research revealed that particle rises were associated with increases in markers of inflammation and toxicological studies showed mechanisms consistent with the hypothesis [29]. It is becoming apparent that particle numbers, which reflect best the ultrafines, may be associated with cardiac effects whereas particle mass is more clearly associated with respiratory end points [30], which is what one would expect since the larger particles which contribute more to mass are deposited on the airways [31]. The mechanisms of action of ultrafine particles on the heart are still the subject of active investigation, but my hunch is that they influence inflammation, endothelial stickiness and fibrinogen activation [32], a threefold attack on coagulation. In this they would be acting rather like bacterial infections.

The final part of the story came in 2003 when I was asked to join a working group of the Royal Society and Royal Academy of Engineering on nanoscience and nanotechnology. I learned something of these new and potentially disruptive technologies and my role was to attempt to foresee any possible health hazards that might arise in their development and applications. We considered not only direct hazards to human health but also hazards that might arise from environmental disruption. In our report we concentrated on two main issues - one arising from our understanding of particulate air pollution and the other from experience with asbestos. We understood there to be many applications of nanotechnologies that promised only benefits to society but that, in the area of the manufacture and use of nanoparticles there were foreseeable hazards [33]. First, if individuals were exposed in the air or on the skin to large numbers of nanoparticles they might have effects, through absorption, on distant organs as well as on the one to which they were applied. For example, inhalation might affect the heart or the brain, the latter possibly through neural transmission along the olfactory nerve. These possible effects would of course depend on dose but also on the surface properties of the particle, which could even be engineered to be beneficial. Secondly, particles shaped like asbestos, if persistent in tissue and of appropriate length, could cause the same effects as that mineral. There is a fundamental difference to classical concepts of toxicology here. Usually we think of effects being a consequence of dose to the target organ, but with nanoparticles we have also to think in terms of effects on target cells or organs causing secondary effects on other organs. This point has not yet been grasped by everyone working in the field. Our report proved influential, and it is probably reasonable to say that it initiated the current interest in nanotoxicology [34,35].

## Conclusions

Over 50 years I have learnt many things, of which the most important are:

Scientists gain much by working in a multi-disciplinary environment;

Science progresses mainly by lateral leaps in thinking, often provoked by chance reading or sceptical questioning of accepted wisdom;

Medicine is no more nor less than human ecology, and humans (and their genomes) have been shaped by evolution in their own particular environments;

Toxicology and epidemiology are indispensible partners in understanding the causation of disease, and many research opportunities exist in the interface of the two.

Everything is made of particles and the study of particles is interesting!
